# A Rare Case of Recurrent Arterial Thrombosis Secondary to Iron Deficiency Anemia

**DOI:** 10.7759/cureus.22117

**Published:** 2022-02-11

**Authors:** Hira Aslam, Ali N Khan, Ahmed Jamal Chaudhary, Sana Iqbal, Rana Ismail

**Affiliations:** 1 Internal Medicine, Detroit Medical Center/Wayne State University (DMC/WSU) Sinai Grace Hospital, Detroit, USA

**Keywords:** thrombotic complications, venous thrombosis, arterial thrombosis, reactive thrombocytosis, iron deficiency anemia (ida)

## Abstract

Iron deficiency anemia is the leading cause of anemia all over the world. Iron deficiency is known to cause reactive thrombocytosis. However, arterial thrombosis secondary to reactive thrombocytosis is a rare entity. In this article, we present a case of a 37-year-old female with recurrent arterial thrombosis due to severe thrombocytosis caused by iron deficiency anemia. The patient developed spleen and kidney infractions, as well as abdominal aortic thrombosis. She was subsequently treated with iron and aspirin with an improvement of the anemia and thrombocytosis, with no further thrombotic complications. Arterial thrombosis is a very serious condition as the thrombus can embolize to carotid arteries leading to stroke or to peripheral blood vessels causing peripheral ischemia and gangrene. Iron deficiency anemia is a reversible cause of thrombocytosis that can be treated very easily to avoid thrombotic complications.

## Introduction

Approximately 10 million people in the United States have iron deficiency, and 5 million people have iron deficiency anemia [1]. Iron deficiency anemia causes reactive thrombocytosis and is known to increase the risk of thrombocytosis by fourfold to fivefold [2]. This increased platelet count is responsible for thrombosis in the setting of an iron-deficient state. While thrombosis is a common complication of essential or primary thrombocythemia, reactive or secondary thrombocytosis is hardly associated with significant thrombotic complications [3]. In addition, there have been far more cases of venous thromboembolism in patients with iron deficiency anemia in the literature compared to arterial thrombosis. Herein, we present a rare case of recurrent arterial thrombosis secondary to severe iron deficiency anemia. 

## Case presentation

**Figure 1:**
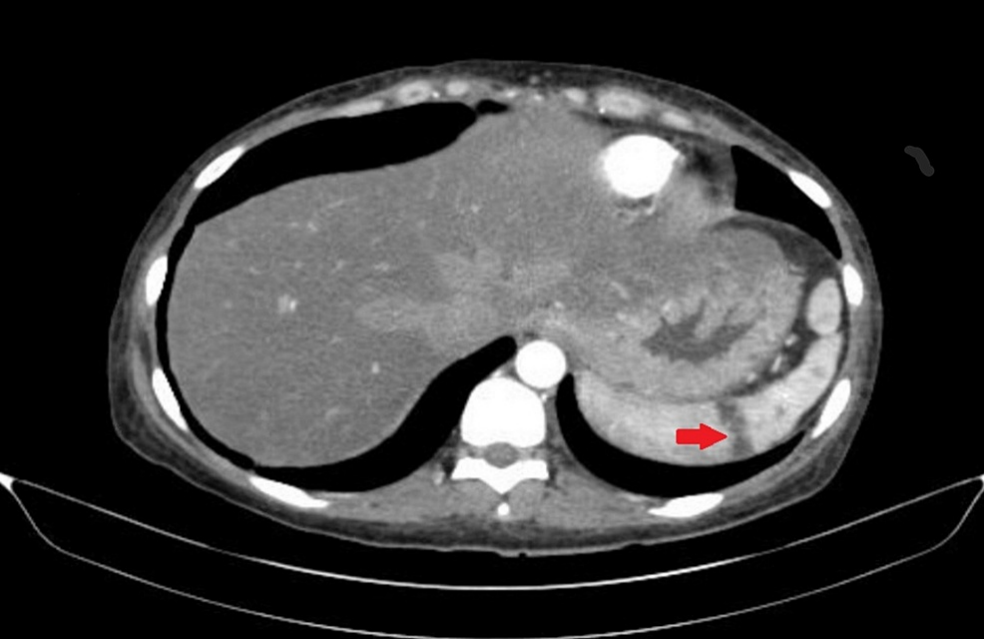
CT abdomen showing wedge-shaped splenic infarct.

A 37-year-old female with fibroid uterus and iron deficiency anemia presented with right upper quadrant abdominal pain and one episode of non-bloody, non-bilious emesis. She also complained of chronic fatigue. The patient denied any fever, jaundice, and nausea. On examination, the patient was vitally stable with blood pressure 141/82 mmHg, heart rate 97 beats per minute, respiratory rate 16 breaths per minute, oral temperature 36.4 degrees Celsius, and saturating 100% on room air. An abdominal exam elicited tenderness in the right hypochondriac as well as the right lumbar region. No organomegaly, abdominal distension, or mental status changes were noted. A year ago, the patient had presented with a similar complaint of left upper quadrant abdominal pain and tenderness. At that time, the CT abdomen showed a 2.1 cm x 1.6 cm hypodense wedge-shaped area in the spleen, likely an infarct (Figure 1). Of note, the patient had a platelet count of 544 x 109/L and a hemoglobin level of 7.8 g/dL at that time. Urinalysis was positive for urinary tract infection, for which she received antibiotics. Splenic infarct was considered an incidental finding, and the patient was discharged.

**Figure 2:**
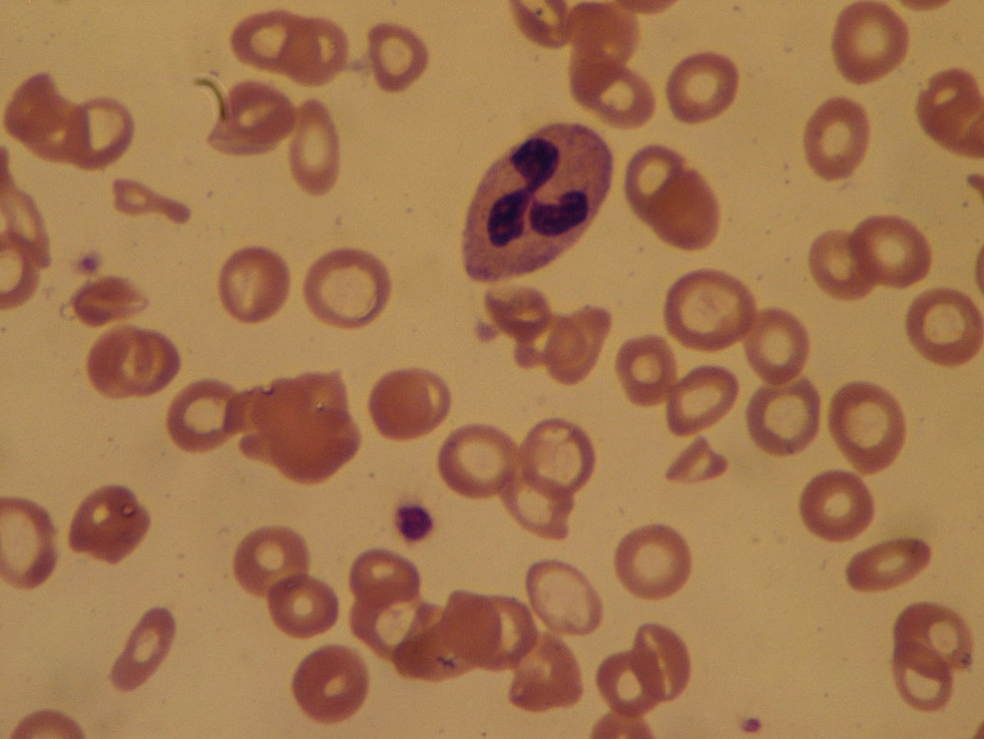
Peripheral smear showing typical features of iron deficiency anemia.

**Figure 3:**
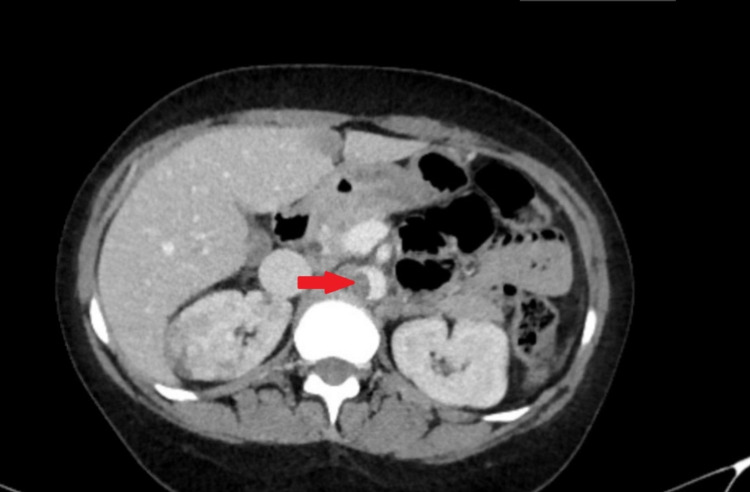
CT abdomen showing thrombus along the wall of abdominal aorta.

**Figure 4:**
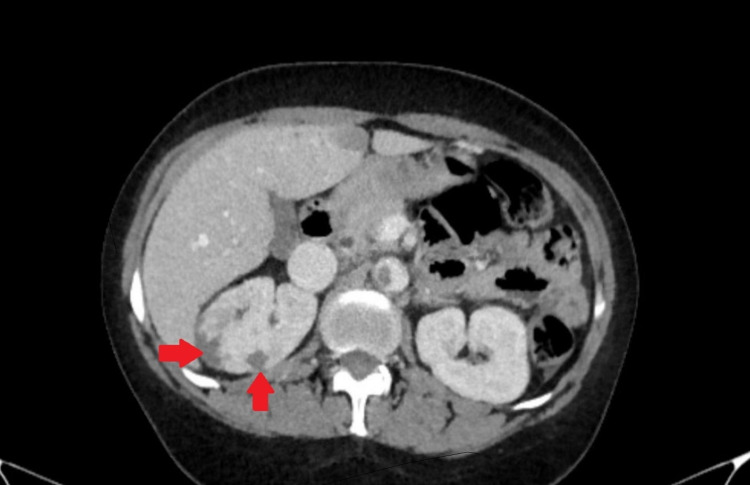
CT abdomen showing wedge-shaped hypodensities in right kidney.

On this admission, laboratory workup showed a hemoglobin level of 4.0 g/dL, hematocrit 15.8%, mean corpuscular volume (MCV) 64.0 FL, red cell distribution width (RDW) 36.9%, white blood cell (WBC) count 18.8 x 103/mm3, and platelet count of 1173 x 109/L. Iron studies showed an iron level of <10 ug/dL, an unsaturated iron-binding capacity of 674 ug/dL, and a ferritin level of 4.2 ng/mL, which established the diagnosis of severe iron deficiency anemia. Peripheral blood smear showed elevated platelet count, microcytosis, and hypochromasia (Figure 2). The patient’s iron deficiency anemia was attributed to chronic bleeding secondary to uterine fibroids. The CT scan of the abdomen was performed, which showed a thrombus along the abdominal aortic wall, projecting into the aortic lumen (Figure 3). It also demonstrated wedge-shaped hypodensities in the right kidney (Figure 4), compatible with renal infarction secondary to the thromboembolic phenomenon caused by an obstructive intramural thrombus in the suprarenal abdominal aorta.

Further hematologic workup to rule out primary thrombocytosis included testing for Janus kinase 2 (JAK2) mutations, which came back negative. The treatment plan consisted of 81 mg aspirin and 325 mg ferrous sulfate daily with regular follow-up. Post-discharge, the patient has been following up regularly in the ambulatory clinic and showing gradual improvement in symptoms and laboratory findings. Her last hemoglobin level was 10.1 g/dL, hematocrit 36.1%, WBC count 7.7 x 103/mm3, and platelet count 227 x 109/L. There have been no reports of further thrombotic complications.

## Discussion

Our patient presented with fatigue and abdominal pain due to severe iron deficiency anemia from menorrhagic uterine fibroids and had elevated platelet count indicating reactive thrombocytosis. The CT scan detected abdominal aortic thrombus and renal and splenic infarcts, which contributed to her presenting chief complaint of abdominal pain. Iron deficiency causes expansion of megakaryocyte progenitors and increases megakaryocyte differentiation [4], thus resulting in platelet count elevation. In this case, the increased platelet count designates the phenomenon known as reactive (or secondary) thrombocytosis, a well-recognized but infrequent outcome caused by iron deficiency anemia. Other causes of reactive thrombocytosis include acute blood loss, acute infection, asplenia, cancer, chronic inflammatory, or infectious diseases [5]. Although less common in the context of iron deficiency anemia, it is possible that extreme thrombocytosis (platelet count of more than 1000 x 109/L) induced a platelet coagulable activity [6] and caused thrombosis in this patient. The exact pathophysiological mechanism is not well understood.

A population-based study by Hung et al. (2015) showed that patients with prior iron deficiency anemia are at an increased risk of venous thromboembolism [7]. According to another large case series, cerebral thrombosis comprised 70.4% of thrombosis cases in patients with iron deficiency anemia, of which 60% were venous [8]. Although iron deficiency and reactive thrombocytosis are widely associated with venous thrombosis, few arterial thrombosis cases have been reported. A prospective study on 108521 patients with high platelet counts in Copenhagen showed an increased risk of cerebral arterial thrombosis by 1.8-fold [9]. Similarly, a large study examining thrombotic complications in a database query of 36327 patients with iron deficiency anemia showed that 32.6% developed reactive thrombocytosis, and only 7.8% had thrombosis. However, the thrombotic risk increased two-fold compared to patients with iron deficiency anemia without thrombocytosis, and the events doubled to 15.8% in patients who had thrombocytosis and iron-deficiency anemia, with respective venous and arterial thrombosis event rates of 14.4% and 1.4% [10]. Multiple stroke cases secondary to iron deficiency anemia and reactive thrombocytosis have been described in the literature [11]. In addition, another case report on three women with menorrhagia who developed carotid artery thrombus -- with no evidence of atherosclerosis -- linked its occurrence to a state of severe iron deficiency anemia and thrombocytosis [12].

Arterial thrombosis usually occurs secondary to atherosclerotic plaque rupture. However, thrombus formation in this patient in the absence of atherosclerotic plaque points towards thrombocytosis and increased platelet coagulant activity as a prothrombotic risk factor for arterial thrombosis. In addition to stroke, reactive thrombocytosis secondary to iron deficiency anemia can also lead to peripheral vascular disease and gangrene. Moreover, a high risk of venous thromboembolism increases the chances of pulmonary embolism.

## Conclusions

Our patient’s symptoms improved after aspirin and ferrous sulfate treatment, and there was no life-threatening thrombotic complication. However, this case report points out that iron deficiency is a reversible cause for thrombocytosis and can be regarded as an indirect risk factor for both arterial and venous thromboembolism. Therefore, patients with concomitant anemia and thrombocytosis should be investigated for iron deficiency and should be treated promptly to prevent the development of thrombotic complications.
